# Considerations for Defining Cytokine Dose, Duration, and Milieu That Are Appropriate for Modeling Chronic Low-Grade Inflammation in Type 2 Diabetes

**DOI:** 10.1155/2016/2846570

**Published:** 2016-10-23

**Authors:** Craig S. Nunemaker

**Affiliations:** ^1^Diabetes Institute, Heritage College of Osteopathic Medicine, Ohio University, Athens, OH, USA; ^2^Department of Biomedical Sciences, Heritage College of Osteopathic Medicine, Ohio University, Athens, OH, USA

## Abstract

Proinflammatory cytokines have been implicated in the pathophysiology of both type 1 diabetes (T1D) and type 2 diabetes (T2D). T1D is an autoimmune disease involving the adaptive immune system responding to pancreatic beta-cells as antigen-presenting cells. This attracts immune cells that surround pancreatic islets (insulitis) and secrete cytokines, such as IL-1beta, IFN-gamma, and TNF-alpha, in close proximity to pancreatic beta-cells. In contrast, there is little evidence for such a focused autoimmune response in T2D. Instead, the innate immune system, which responds to cellular damage and pathogens, appears to play a key role. There are three major sources of proinflammatory cytokines that may impact islet/beta-cell function in T2D: (1) from islet cells, (2) from increased numbers of intraislet macrophages/immune cells, and (3) from increased circulating levels of proinflammatory cytokines due to obesity, presumably coming from inflamed adipose tissue. These differences between T1D and T2D are reflected by significant differences in the cytokine concentration, duration, and milieu. This review focuses on chronic versus acute cytokine action, cytokine concentrations, and cytokine milieu from the perspective of the pancreatic islet in T2D. We conclude that new cytokine models may be needed to reflect the pathophysiology of T2D more effectively than what are currently employed.

## 1. Introduction

Nearly a century has passed since insulin codiscoverers Frederick Banting and Charles Best observed the first patient to receive insulin therapy in 1922, instantly transforming the prognosis of type 1 diabetes (T1D) from a death sentence to a manageable disease. However, the root cause of T1D is still not known, but the processes that lead to the destruction of insulin-producing pancreatic beta-cells have been fairly well elucidated. T1D is now considered an autoimmune disease that causes the gradual, systematic destruction of pancreatic beta-cells within the islets of Langerhans. Proinflammatory cytokines play a prominent role in the pathophysiology of T1D [1–6], but increasing evidence also suggests a significant role for cytokines in islet dysfunction in T2D as well [6–8]. In this review, I address the substantial differences in the inflammatory environment of the pancreatic islet in T1D versus T2D and then consider alternative models of cytokine exposure that may more accurately reflect the pancreatic environment in T2D, with particular emphasis on the islet.

## 2. Cytokines in T1D versus T2D

**Figure 1:**
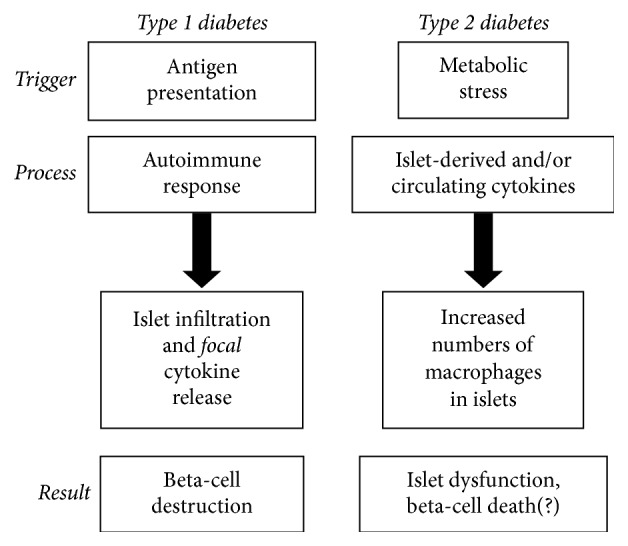
A flowchart of very basic differences in the pathophysiology of T1D and T2D. Each disease is described by the immune-mediated triggers, processes, and results with respect to the pancreatic beta-cell/islet.

There are notable similarities and differences in the action of cytokines in the development of T1D versus T2D [9, 10], as shown in Figure 1 and described below. In T1D, beta-cells are the direct target of an autoimmune invasion beginning with peri-insulitis and ending in beta-cell death [10–12]. In T2D, metabolic stress is thought to activate the innate immune system, resulting in a chronic inflammatory state marked by increased cytokines, increased islet-associated macrophages, and beta-cell apoptosis [10, 13, 14]. In T1D, autoimmunity is the prime effector of beta-cell destruction. In T2D, a number of metabolic stress factors related to excess nutrients are thought to contribute to beta-cell decline and destruction including glucotoxicity [15–17], lipotoxicity [15–17], oxidative stress [16, 18], endoplasmic reticulum (ER) stress [19, 20], amyloid deposition [21], and inflammation [8, 13]. Some evidence suggests that all of these factors may even be connected through inflammasome activation [22–24]. To bring this discussion full circle, emerging research suggests that some of the putative factors causing beta-cell dysfunction in T2D, most notably ER stress and/or oxidative stress, may play a role in antigen presentation to trigger the autoimmune response in T1D [25–27].

The key proinflammatory cytokines in T1D are interleukin- (IL-) 1beta, tumor necrosis factor alpha (TNF-alpha), and interferon-gamma (IFN-gamma). IL-1beta is produced by monocytes (leukocytes), macrophages, and other immune cells. TNF-alpha is produced by macrophages, lymphoid, stromal cells, and many other cell types and can exist in membrane-bound as well as in soluble forms [28, 29]. IFN-gamma is primarily produced by natural killer cells and certain types of T cells [30], although macrophages can also produce IFN-gamma under certain conditions, such as exposure to IL-12 and IL-18 [31, 32]. Infiltrating immune cells are thought to secrete these three cytokines in close proximity to the beta-cell at high concentrations in T1D, and these cytokines interact synergistically to inflict cytotoxic effects [3, 33–36]. Although rodent islets may succumb to high concentrations of any of these cytokines, classic studies demonstrated that combinations of these cytokines produced more consistent cytotoxic effects on human islets [3, 4, 34, 37, 38]. In T2D, the metabolic stress of obesity is thought to elevate cytokine production in adipose tissue [39–43] and other organs to a lesser extent [44], resulting in an overall chronic low-grade inflammatory state. In the next section, key parameters will be examined to define a model of islet exposure to cytokines that may resemble the pancreatic environment more closely during the development of T2D.

## 3. Considerations for a Model of Cytokine Action Consistent with T2D

**Figure 2:**
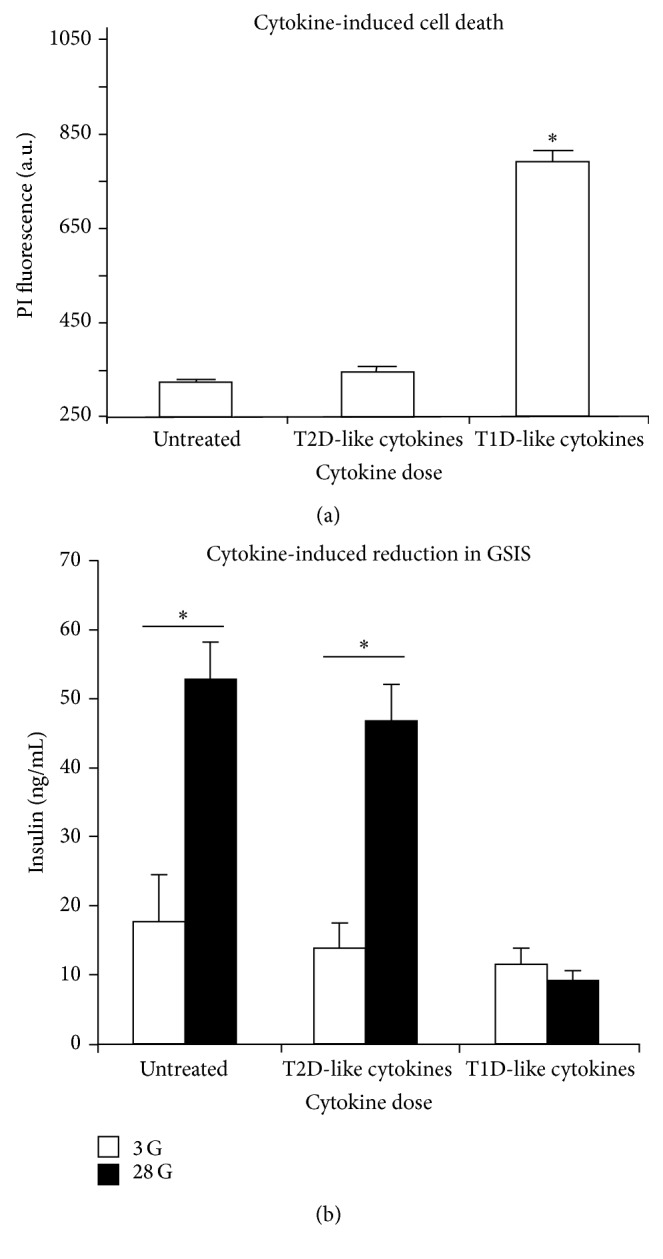
Islet viability and function following overnight treatment with different cytokine concentrations. (a) Cell death measurements by propidium iodide (PI) were made on islets following treatment with Roswell Park Memorial Institute 1640 media (untreated), 10 pg/mL IL-1beta + 20 pg/mL TNF-alpha + 200 pg/mL IFN-gamma (T2D-like), or 500 pg/mL IL-1beta + 1000 pg/mL TNF-alpha + 10,000 pg/mL IFN-gamma (T1D-like). (b) Glucose-stimulated insulin secretion (GSIS) in 3 mM glucose (3G, open bars) and 28 mM glucose (28G, filled bars) following cytokine treatments listed in (a). ^*∗*^ *P* value < 0.05.

A number of proinflammatory cytokines are elevated in the general circulation of obese individuals compared to lean individuals, and these increased levels are associated with an increased risk of developing T2D [45–53]. This low-grade systemic inflammation may also play a direct role in triggering beta-cell dysfunction, particularly in T2D. We have previously reported that cytokines can directly affect aspects of islet function in rodent islets at circulating concentrations in vitro [54–56]. However, these effects, which are primarily related to intracellular calcium handling, do not appear to impact cell death rate or insulin secretion in normal islets [55]. As shown in Figure 2, the “T2D-like” cytokines, in the range of circulating levels in the blood, do not increase cell death or decrease insulin secretion significantly. The “T1D-like” concentrations of cytokines necessary to induce cell death are at least 50-fold higher than what is observed in low-grade systemic inflammation in T2D (see also similar published data in [55]). The 5 ng/mL concentration of IL-1beta commonly found in the literature is 10 times greater than the T1D-like concentration and 500 times greater than the T2D-like concentration shown in Figure 2. This suggests that the use of high concentrations of death-inducing cytokines is not necessarily appropriate for recreating an islet environment related to T2D. To build an appropriate in vitro model of chronic low-grade inflammation that can reasonably mimic the islet environment in T2D in vivo, several questions must be addressed:

1. What cytokines and chemokines are elevated in the systemic circulation in obesity and T2D that can negatively impact islet function?
2. What immune cells are found in or near islets that can secrete cytokines in close proximity?
3. What cytokines are secreted by islet cells themselves in response to damage or stress?
4. What concentration and duration of exposure mimic chronic low-grade inflammation?

Each of these considerations is discussed in Sections 3.1–3.4.

### 3.1. Circulating Cytokines and Chemokines

Circulating levels of several cytokines and chemokines have been identified as potential risk factors for developing T2D, including TNF-alpha, C-reactive protein (CRP), monocyte chemoattractant protein-1 (MCP-1), IL-1beta, and IL-6 [45–53]. Chief among these risk factors are IL-1beta and IL-6 [57]. IL-1beta at high enough concentrations can destroy beta-cells [58, 59]. Antagonizing the actions of IL-1beta has been shown to have clinical value in partially mitigating the symptoms of T2D [60–63], suggesting that IL-1beta plays a role in the pathophysiology of the disease. Numerous studies have also associated increased levels of IL-6 with the development of type 2 diabetes [64–67]. However, IL-6 has also been associated with beneficial effects of exercise (reviewed in [68]) and with improving islet function [69, 70], resulting in a complex and evolving view over the role(s) of IL-6 in T2D [71–73]. A prospective study focused primarily on links between nutrition and cancer generated an intriguing report that identified the combination of both IL-1beta and IL-6 as key cocontributors to the development of T2D [47]. We reported that serum levels of IL-1beta and IL-6 in diabetes-prone mice at an age before hyperglycemia developed were 2-3 times higher than for age-matched heterozygous control mice, suggesting that low-grade systemic inflammation develops early in the disease process [74].

In addition, many other cytokines and chemokines are increased in obesity including leptin, resistin, IL-7, IL-8, retinol binding protein- (RBP-) 4, plasminogen activator inhibitor- (PAI-) 1, chemokine- (C-X-C motif-) ligand 5 (CXCL5), visfatin, chemerin, and vaspin [75, 76]. Other cytokines are decreased with increased obesity including adiponectin, IL-10, and omentin [75, 76]. We conducted a 32-plex cytokine detection array of mouse blood serum from leptin-receptor-deficient male BKS.Cg-Dock7m+/+ Leprdb/J (db/db) mice and heterozygous controls at different ages to identify additional cytokines and chemokines [77]. Many of the 32 cytokines tested were above or below the sensitivity for appropriate detection or were highly variable. However, CXCL1 and CXCL5 were increased significantly in serum at the onset of obesity and T2D. We further showed that exposure to circulating concentrations of these chemokines synergistically produced mild inhibition of islet function and that expression of CXCL1 and CXCL5 increased markedly in islets in response to low-grade inflammation [77].

Regarding the classic trio of T1D cytokines, IL-1beta appears to also play a role in T2D, but TNF-alpha and IFN-gamma may or may not be as prominent. In our studies, we did not observe elevated serum levels of TNF-alpha in prediabetic mice (IFN-gamma was not examined), and TNF-alpha does not appear to have the same degree of impact as IL-1beta and IL-6 on the development of T2D in humans [47]. IFN-gamma appears to be elevated in obesity and may play a role in islet dysfunction in T2D [78, 79]. However, at present there is not sufficient evidence that circulating levels of IFN-gamma impact islet function or that IFN-producing immune cells are localized within islets in models of T2D. Additional studies are needed to determine the full milieu of obesity-associated cytokines and the extent to which this milieu of circulating cytokines can impact islet function.

### 3.2. Cytokines Produced by Macrophages and Other Immune Cells within Islets

In addition to the chronic inflammatory state marked by increased circulating cytokines, signs of increased inflammation in T2D have also been observed within the pancreas itself. Specifically, increased numbers of islet-associated macrophages have been reported in rodent models of T2D and in humans with T2D [80, 81]. In the db/db mouse model of diabetes, a 4-fold increase in M1 type macrophages was reported as early as 8 weeks of age [82]. Macrophages are involved in adaptation and cellular repair as well as in the discrete removal of dead and dying cells. Thus, increased islet presence of macrophages could merely be an indicator of more dying cells that must be cleared away. However, macrophages could also be active instigators of destruction by secreting various proinflammatory cytokines in close proximity to islet cells. The potential of macrophage instigation of islet dysfunction has been confirmed by studies showing that palmitate causes macrophage accumulation, increased cytokine/chemokine production within islets, and islet dysfunction, but islet dysfunction is prevented when macrophage accumulation is blocked [83, 84]. These findings suggest that macrophages play an important role in inflammation caused by metabolic stress. In addition to macrophage involvement, recent work also suggests that clusters of differentiation- (CD-) 45-positive leukocytes are also increased in islets from T2D donors, suggesting a possible role of the adaptive immune system in T2D [85, 86].

### 3.3. Islet-Derived Cytokines

There is also evidence that cells within islets are capable of producing proinflammatory cytokines. Chronic high levels of glucose, for example, can stimulate IL-1beta production in beta-cells [87]. Exposure to certain cytokines can also stimulate islet expression of cytokines and chemokines. For example, treatment with IFN-gamma + TNF-alpha increases islet expression of IL-15, interferon-gamma-induced protein- (IP-) 10, CXCL9, CXCL11, and chemokine (C-C motif) ligand- (CCL-) 20 [88]. IL-1beta exposure induces expression of several neutrophil-attracting proteins including MCP-1 [89]. Exposure to free fatty acids, palmitate in particular, can induce islet expression of numerous cytokines and chemokines, including IL-1beta, TNF-alpha, IL-6, IL-8, CXCL1, and MCP-1 [83, 84, 90].

The importance of intraislet cytokine production is that cytokine concentrations within islets may be orders of magnitude higher when cytokines are produced by islet cells and/or resident immune cells within the islet in comparison to circulating levels. It should be noted that the increased cytokine expression in many of these studies could not be attributed to any specific cell type(s) within the islet. Thus, it is possible that immune cells within the islet could be responsible for most or all of the increased expression of these cytokines, rather than insulin-producing beta-cells, glucagon-producing alpha cell, or other endocrine cell types in the islet. Thus, intraislet secretion of cytokines, regardless of the source, may markedly increase the potential concentration of cytokine exposure in islet cells.

### 3.4. Cytokine Dose, Duration, and Milieu

The net effect of cytokine exposure on tissue depends markedly on factors such as cytokine concentration, duration of cytokine exposure, and the combination of cytokines involved (also called the cytokine milieu [91, 92]). IL-1beta, for example, causes islet dysfunction or cell death in a number of studies of chronic exposure [37, 58], and blockade of the IL-1 receptor mitigates many of these effects [60, 93, 94]. However, IL-1beta can also enhance insulin secretion [95–97], and recent work even suggests IL-1beta may play a key role in islet compensation for nutrient overload in the early stages of metabolic disorders [98]. In one early series of studies, both positive and negative impacts of IL-1beta were described based on differences and dose and duration of exposure [99–101]. In the context of T2D, the duration of low-grade inflammation may be a key factor, as cytokines could have compensatory/protective effects initially that become deleterious under chronic conditions. Thus, the dose and duration of cytokine exposure [98, 101, 102], species being tested [103–105], and milieu of interacting cytokines [3, 4, 95, 105, 106] are all important factors that contribute to both positive or negative effects on the beta-cell. In addition, synergistic activity among multiple cytokines can alter or amplify signaling pathways [3, 4, 107, 108], adding an additional layer of complexity to cytokine action.

## 4. Cytokine Susceptibility and Signaling in T2D-Prone Islets

Our recent work has focused on elucidating possible differences in response to chronic low-grade inflammation between islets from diabetes-prone and normal (nondiabetes-prone) environments. Male db/db mice and heterozygous controls were used at 4-5 weeks of age, an age at which the capacity for glucose-stimulated insulin secretion and intracellular calcium responses are similar for both mouse strains [74]. The combination of IL-1beta and IL-6 significantly impaired islet function in ways that either alone could not [74]. Further, islets isolated from prediabetic db/db mice and exposed in vitro to IL-1beta + IL-6 overnight at approximately circulating levels (in mice) caused a significant decrease in glucose-stimulated insulin secretion, a significant increase in ER stress markers (nitric oxide synthase 2 (NOS2), 78-kDa glucose-regulated protein (GRP78), activating transcription factor 4 (ATF4), and DNA Damage Inducible Transcript 3 (DDIT3), also known as C/EBP homologous protein (CHOP)), and increased cell death; these cytokines produced no such effect in heterozygous control islets [74]. Further, when nondiabetic control mice were implanted with subcutaneous osmotic mini-pumps containing IL-1beta + IL-6 to mimic the serum increases found in prediabetic db/db mice, we were able to produce 2-3-fold increases in circulating cytokines, thus reproducing cytokine levels observed in low-grade inflammation in obesity. The increased circulating levels of IL-1beta + IL-6 were insufficient to impact physiology in these normal mice. When compared with saline-implanted controls, however, isolated islets from cytokine-pump mice showed deficiencies in calcium handling and insulin secretion that were similar to cytokine effects on islets in vitro [74]. Finally, these low level cytokine exposures could also impair human islet function [74]. These results in mice suggest that mild increases in circulating cytokines may be sufficient to trigger islet dysfunction leading to islet failure in genetically susceptible individuals [74].

The cytokine signaling pathways may also differ in conditions of low-grade inflammation when compared to classic cytokine cocktails associated with T1D. A microarray study of islet gene expression in response to low concentrations 10 pg/mL IL-1beta + 20 pg/mL IL-6 produced a large number of gene hits that were apparently not associated with canonical signaling pathways for IL-1beta and IL-6 [109]. Several highly cytokine-induced genes related to proteins involved with iron regulation, including Steap4 (six-transmembrane epithelial antigen of prostate 4), lipocalin-2 (Lcn-2), and hepcidin antimicrobial peptide (Hamp). Moreover, seven cytokine-sensitive genes were identified for single nucleotide polymorphisms related to the acute insulin response to glucose (AIRg, a test of islet function) in a genome-wide association scan of a population with a high prevalence for T2D [109]. The seven genes were Arap3 (ArfGAP with RhoGAP domain, ankyrin repeat, and PH domain 3), F13a1 (coagulation factor XIII, A1 polypeptide), Klhl6 (kelch-like protein 6), Nid1 (nidogen 1), Pamr1 (peptidase domain-containing protein associated with muscle regeneration 1), Ripk2 (receptor-interacting protein kinase 2), and Steap4 [109]. These findings suggest novel elements of cytokine signaling that require further study.

## 5. Modeling Inflammation in T2D

**Figure 3:**
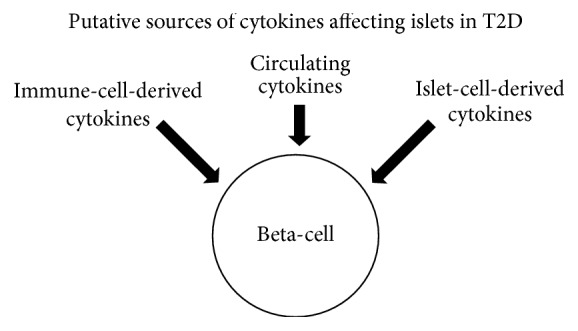
A basic depiction of the putative sources of cytokines affecting islets in T2D.

The microenvironment of the pancreatic islet during the development of T2D has yet to be fully elucidated. With regard to inflammatory-mediated processes, several sources of proinflammatory cytokines have been suggested throughout this review: (1) immune-cell-derived cytokines from macrophages and lymphocytes, (2) cytokines derived from inflamed fat tissues or other distal sources that increase cytokine concentration in the general circulation, and (3) cytokines/chemokines derived from peptide-producing islet cells such as alpha-cells or beta-cells (see Figure 3). Creating accurate in vitro models of the concentration, duration, and combination of key cytokines involved with each of these sources will contribute greatly toward a better understanding of how islets and other organs such as the liver, muscle, fat, and kidney respond to low-grade inflammation.

We have made a first attempt at a model of the circulating levels of proinflammatory cytokines utilizing IL-1beta and IL-6. Although the inflammatory environment is dynamic, we focused on cytokines involved prior to the onset of hyperglycemia (the prediabetic stage). Several observations served as our rationale for developing this model of circulating cytokines in T2D. First, the combination of these two cytokines is sufficient to significantly increase the risk of developing T2D in humans [47]. Second, increased levels of IL-1beta and IL-6 were observed in db/db mice prior to hyperglycemia or substantial differences in weight (<10%) compared to control mice [74]. Differences in several other cytokines, including TNF-alpha [74], were not observed [77]. Third, exposure to combinations of these cytokines at circulating levels affected islets in unique ways that treatment with individual cytokines could not replicate [55, 74]. These studies collectively point to the combination of 5–10 pg/mL IL-1beta and 10–20 pg/mL IL-6 as being sufficient to promote islet dysfunction in T2D.

To produce these levels of low-grade systemic inflammation in vivo is quite possible. Osmotic mini-pumps are ideally designed to produce the small and precise changes in the circulating levels of cytokines that define chronic inflammation. We induced low-grade inflammation in vivo in normal healthy mice loading ALZET osmotic mini-pumps (model 1007d, rate 0.5 *μ*L/h for 7 days) with 32 *μ*g/mL for IL-1B plus 4 *μ*g/mL for IL-6 in saline or saline only controls. We inserted each pump subcutaneously into an incision at the nape of the neck to cause low-grade inflammation in mice without significantly impacting blood glucose or insulin levels. ELISA assays confirmed that serum levels of IL-1B and IL-6 were approximately doubled in mice treated with cytokine mini-pumps versus saline control pumps. Islets isolated from cytokine-pump-treated mice showed impaired function compared to saline-pump controls. These findings are detailed in [74] and provide a method for elevating specific circulating cytokines related to chronic low-grade inflammation in T2D, independent of obesity.

## 6. Final Thoughts

Differences in susceptibility to cytokine-induced damage between diabetes-prone and nondiabetes-prone islets suggest that certain genetic predispositions may render some individuals much more susceptible to the negative impact of proinflammatory cytokines than others. How many other chronic diseases could play out in this way? The relatively small increases in circulating cytokines caused by obesity or other chronic inflammatory conditions could conceivably contribute to a plethora of conditions including asthma [110, 111], rheumatoid arthritis [112], heart disease [113], various cancers [114–116], polycystic ovarian syndrome [117, 118], and even mood disorders [119]. Thus, the combination of the genetic predisposition of the individual and the dose, duration, and milieu of cytokine exposure may significantly contribute to numerous chronic diseases.
